# Evaluation of Autologous Serum Skin Test and Skin Prick Test Reactivity to House Dust Mite in Patients with Chronic Spontaneous Urticaria

**DOI:** 10.1371/journal.pone.0064142

**Published:** 2013-05-31

**Authors:** Zhiqiang Song, Zhifang Zhai, Hua Zhong, Ziyuan Zhou, WenChieh Chen, Fei Hao

**Affiliations:** 1 Department of Dermatology, Southwest Hospital, The Third Military Medical University, Chongqing, China; 2 Department of Dermatology and Allergy, Technische Universitaet Muenchen, Munich, Germany; 3 Department of Toxicology, Preventive College, The Third Military Medical University, Chongqing, China; Cardiff University, United Kingdom

## Abstract

**Background:**

Chronic spontaneous urticaria (CSU) is a common skin disorder with etiology that is not well understood.

**Methods:**

In this study, we evaluated the prevalence of autologous serum skin test (ASST) and skin prick testing (SPT) to house dust mite (HDM) in 862 CSU cases in China. Clinical features, courses and treatment responses were also recorded.

**Results:**

The prevalence of positive ASST was 46.3%, and patients aged 30–39 years had the highest positive rate (52.1%). Positive SPT to HDM was seen in 153 patients (17.7%) with the highest positive rate (34.2%) in patients aged 20 or less. Patients with positive ASST had higher urticaria activity scores (UAS) (4.18±0.65 *vs.* 3.67±0.53) but lower positive rates of HDM (24.6% *vs.* 37.6%), as compared with those with negative ASST (odds ratio (OR) 1.84, 95% CI 1.38–2.47). Patients could be categorized into four groups based on the results of ASST and SPT to HDM and patients with positive ASST and positive SPT to HDM had the highest disease activity scores, experienced higher frequencies of angioedema, diseases duration, and required higher dosage of loratadine every month, compared with other subgroups (*P*<0.0001).

**Conclusions:**

Patients with CSU showed varied responses of positive ASST and varied sensitivity to HDM, Patients with positive ASST and/or positive SPT had more disease activity compared with patients with negative ASST and/or negative SPT. Further classification can be made based on the result of SPT and ASST.

## Introduction

Chronic urticaria (CU) is a common skin disorder affecting 15–20% people in the general population. Based on its duration, frequency and causes, CU can be classified into three clinical subgroups, spontaneous (80%), physical (10%) and special forms (10%) [1]. Chronic spontaneous urticaria (CSU), also known as chronic idiopathic urticaria, is characterized by spontaneous occurrence of wheals without an obvious stimulus lasting for more than 6 weeks [1], [2]. Pathogenesis of CSU is unclear and possible causes may include chronic infections, allergy to certain food or food additives, anxiety, and autoantibody production against immunoglobulin E (IgE) receptor [1], [2], [3], [4], [5], [6].

Many studies have shown that up to 30–50% patients with CSU are associated with autoimmune manifestations, among which 35–40% presents functional mast cell stimulating IgG antibodies against the α chain of the high-affinity IgE receptor, with 5–10% of patients producing antibodies towards IgE itself [1], [3], [7], [8], [9], [10], [11], [12]. Meanwhile, a number of studies have shown that between 25–58% of CU patients had a positive cutaneous reaction and/or evaluated serum specific IgE antibodies against house dust mite (HDM), suggesting a possible association between HDM sensitivity and a subgroup of CU [13], [14], [15], [16], [17], [18], [19], [20]. However, the prevalence of sensitization to HDM and the positive rate of ASST vary in different studies from different countries.

In this study, we analyzed the clinical and laboratory results from patients with CSU in department of dermatology, Southwest Hospital, Chongqing from January 2007 to December 2011. Reports from our lab, as well as reports by other groups, support that the most common positive aeroallergen in CSU is HDM [14], [15], [17], [18], [19], [20], [21]. Our aims were to explore the prevalence of ASST and sensitization to HDM, and the underlying significances of these two assays in patients with CSU.

## Subjects and Methods

### Study design and patient selection

The study was approved by the Human Ethics Committee of Southwest Hospital of Chongqing (The approval number is KY201009), China. The medical records for patients with chronic urticaria at the Department of Dermatology from January 2007 to December 2011 were retrospectively reviewed. Only patients with CSU were included. The demographic data of patients including age, sex, marital status, disease duration and drug intakes were reviewed. All subjects were informed about the process of SPT and ASST and given the written consent prior to the testing.

### Skin prick test (SPT)

SPT was performed by placing a drop of HDM allergen solution (Allergopharma™ Skin Prick Testing Kits, Reinbeck, Germany) on the inner side of forearm. The solution was allowed to enter the skin using a needle. Saline and histamine phosphate (10 mg/mL) were used as negative and positive controls, respectively. Short-acting antihistamine was avoided at least 3 days prior to SPT, and long-acting antihistamines or immunosuppressants were withdrawn at least one month before the test. The skin response was measured at 30 minutes after pricking. The degrees of the positivity in SPT were assessed and graded on a scale of 0, 1+, 2+ and 3+, by comparing the size of wheal (mean wheal diameter, MWD) induced by HDM allergen with that induced by 10 mg/mL histamine solution [13]. Grade 0 = the allergen-induced mean wheal diameter (aiMWD) is smaller than 1/2 of positive control-induced mean wheal diameter (pciMWD); grade 1 (1+) = aiMWD is equal or greater than 1/2, but smaller than 2/3 of pciMWD; grade 2 (2+) = aiMWD is equal or greater than 2/3 of pciMWD but smaller than pciMWD; grade 3 (3+) = aiMWD is equal or greater than pciMWD. In this study, grade 0 was judged as negative, and grades 1 to 3 were judged as positive.

### Autologous serum skin test (ASST)

Autologous serum (0.05 mL), positive control (10 µg/mL histamine) and negative control (0.3 mg/mL human serum albumin) were separately injected intracutaneously into the flexible forearm with a distance of 5 cm between each injection site. Skin areas that had been involved with spontaneous wheals during the last 24 h were avoided. Antihistamine and immunosuppressant drugs were avoided as described above. The cutaneous responses including wheal and erythema were measured at 30 min after injection. Similarly, cutaneous reactions against autologous serum were assessed and graded on a scale of 0, 1+, 2+ and 3+ by comparing the size of wheal (mean wheal diameter, MWD) induced by autologus serum, with that induced by 10 µg/mL histamine solution. The degree of the positivity in ASST was classified as follows: grade 0 = the serum-induced mean wheal diameter (siMWD) is smaller than 1/2 positive control-induced mean wheal diameter (pciMWD); grade 1(1+) = siMWD is equal or greater than 1/2 but smaller than 2/3 of pciMWD; grade 2 (2+) = siMWD is equal o greater than 2/3 but smaller than pciMWD; grade 3 (3+) = siMWD is equal or greater than pciMWD. In this study, grade 0 was considered as negative, and grades 1 to 3 were considered as positive. This score system showed good coincidence to classic standard of positive judgment in which serum-induced weal had a diameter at least 1.5 mm greater than the HSA-induced wheal in our previous study [22]. ASST and ASST were carried out consecutively in one patient on the same day.

### Detection of serum IgE

Total and HDM-specific IgE levels were measured in serum by using the ImmunoCAP 100 instrument (Pharmacia Diagnostic AB/Thermo Fisher, Uppsala, Sweden) according to the manufacturer's instructions. Specific IgE was considered positive with results of more than 0.35 kU/L.

### Clinical Assessment

Disease activity was evaluated based on urticaria activity score (UAS), which is based on the assessment of key urticaria symptoms (wheals and pruritus) [1], [23]. Briefly, scores for ‘wheal numbers’ (ranged 0–3) and ‘pruritus intensity’ (ranged 0–3) were added together (ranged 0–6). All patients were also asked to complete Dermatology Life Quality Index (DLQI), which comprises 10 equally weighted items that evaluate the effect of skin problems on patients' daily lives: itchiness/soreness/pain; embarrassment; interference with shopping; clothes purchases; social leisure; difficulty playing sports; difficulty with work or study; problems with partner; sexual difficulties; and problems at home caused by treatment. Each item is scored on a continuum from 0 (least impairment) to 3 (worst impairment), with total DLQI scores ranging from 0 to 30 [24]. Trained staff were present to assist the patients in completing the form.

### Statistical analysis

The prevalence of ASST and SPT was determined in whole population as well as in different gender and age-groups (1–19 years, 20–29 years, 30–39 years, 40–49 years, 50+ years). Comparison of groups was performed using the *χ* ^2^-test. Relative risks obtained by logistic regression was expressed in odds ratio (OR) and 95% confidence intervals (95% CI) are given. Differences between variables for two groups were analyzed using unpaired *t*-test or Mann-Whitney *U* test, respectively. The one-way analysis of variance (one-way ANOVA) was performed to detect statistical differences of clinical and laboratory parameters among subgroups. Differences within the ANOVA were determined by using the Tukey's post-hoc test. All statistical data analyses were performed using SPSS for Windows version 13.0 (Chicago,IL, USA). *P* values <0.05 was considered as statistically significant.

## Results

### Patient characteristics

Eight hundred and sixty-two patient (335 males, 527 females; mean age 33.3±9.5 years; ranged 3 to 65 years) diagnosed as CSU were included in this study and they all fulfilled the modified EAACI/GA2LEN/EDF/WAO guideline (spontaneous wheals and/or angioedema >6 weeks) [1]. The mean duration of the disease was 26.4±12.6 months (4–245 months). There were 106 (12.3%) cases that also presented with physical urticaria. 82 (9.5%) patients had a history of atopic diseases, and 59 (6.8%) cases had family history of atopy. The most common atopic diseases were allergic rhinitis (n = 39; 4.5%), atopic dermatitis (n = 17; 1.9%), and asthma (n = 17; 1.9%).

### Autologous serum skin test (ASST)

**Figure 1:**
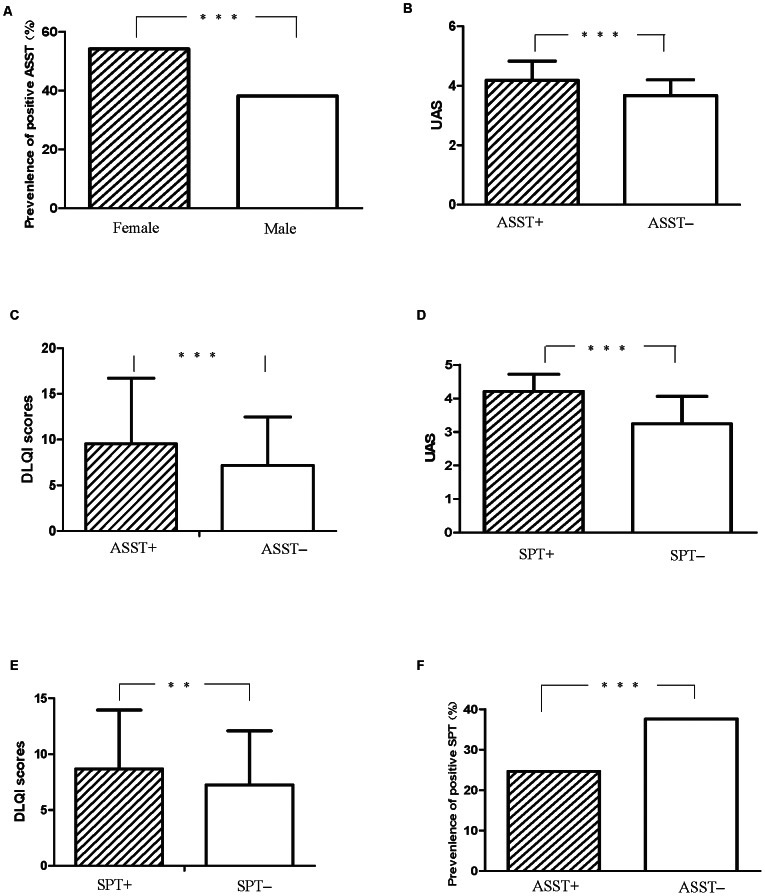
Detection of autologous serum skin test and skin prick test to HDM in 862 patients with chronic spontaneous urticaria. A. Comparison of percent of positive ASST in female patients and male patients. B. Comparison of urticaria activity scores (UAS) between patients with positive ASST and patients with negative ASST. C. Comparison of DLQI scores between patients with positive ASST and patients with negative ASST. D. Comparison of UAS between patients with positive SPT and patients with negative SPT. E. Comparison of DLQI scores between patients with positive SPT and patients with negative SPT. F. Comparison of percent of positive SPT reactions between patients with positive ASST and patients with negative ASST.

A total of 399 patients tested positive in the ASST (46.3%), as shown in Table 1. As for differences among age groups, patients aged from 30–39 had the highest positivity rate (52.1%), followed by patients aged 40–49 (47.2%). Patients aged lower than 20 had the lowest positive rate (33.3%). There were more female patients (51.4%) than male patients (38.2%) with positive ASST (*P* = 0.0002) (Fig. 1A). The OR for having a positive ASST for female relative to male patients was 0.58 (95% CI:0.44 to 0.77). Patients with positive ASST had higher UAS scores, which was statistically significant compared with the ASST-negative group (4.18±0.65 *vs.* 3.67±0.53; *P*<0.0001) (Fig. 1B). Also, the average scores of DLQI in ASST positive subgroup were also significantly higher than that in the ASST-negative subgroup (9.53±7.18 *vs.* 7.2±5.28, *P*<0.0001) (Fig. 1C).

**Table 1:** Result of ASST and SPT to HDM in 862 patients with CSU.

Age groups(yr)	No. of patients	ASST reaction				SPT reaction			
		1+ No. (%)	2+ No. (%)	3+ No.(%)	Total No.(%)	1+ No.(%)	2+ No.(%)	3+ No.(%)	Total No.(%)
≤20	120	7(5.8%)	17(17.2)	16(13.3)	40(33.3)	11(9.2)	13(10.8)	17(14.2)	41(34.2)
21–29	172	15(8.7)	32(18.6)	32(18.6)	79(45.9)	14(8.1)	21(12.2)	15(8.7)	50(29.1)
30–39	259	33(12.7)	52(20.1)	50(19.3)	135(52.1)	11(4.2)	12(4.6)	7(2.7)	30/(11.6)
40–49	195	19(9.7)	46(23.6)	27(13.8)	92(47.2)	8(4.1)	5(2.6)	3(1.5)	16(8.2)
≥50	116	13(11.2)	24(20.7)	16(13.8)	53(45.7)	8(6.9)	6(5.2)	2(1.7)	16(13.8)
Total	862	87(10.1)	171(19.8)	141(16.4)	399(46.3)	52(6.0)	57(6.6)	44(5.1)	153(17.7)

### Skin Prick Test (SPT)

SPT results showed that 153 (17.7%) patients were sensitive to HDM, as shown in Table 1. Although the group of patients aged lower than 20 had the highest positive rate (34.2%), however there was no significant difference among different age groups (*P* = 0.9144). Interestingly, in the follow-up of these SPT positive teenage patients, 13 were diagnosed as atopic diseases, including atopic dermatitis (n = 5,) allergic rhinitis (n = 5) and asthma (n = 3). The positive rate in female patients (25.9%) was similar to that in male patients (26.0%)(*P* = 1.000). The average scores of UAS in SPT-positive subgroup trended higher when compared with the SPT-negative subgroup (4.21±0.51 *vs.* 3.24±0.82; *P*<0.0001) (Fig. 1D). The difference of DLQI between two groups was also statistically significant (8.68±5.27 *vs.* 7.24±4.58; *P* = 0.0002) (Fig. 1E). Furthermore, ASST-positive patients had a lower positive rate of HDM, which was statistically significant compared to that in ASST-negative patients (24.63% *vs.* 37.57%; *P*<0.0001) (Fig. 1F). The OR for having a positive SPT for patients with positive ASST relative to patients with negative SPT was 1.84 (95% CI: 1.38–2.47).

### Clinical characteristics in different subgroups based on the result of ASST and SPT

**Figure 2:**
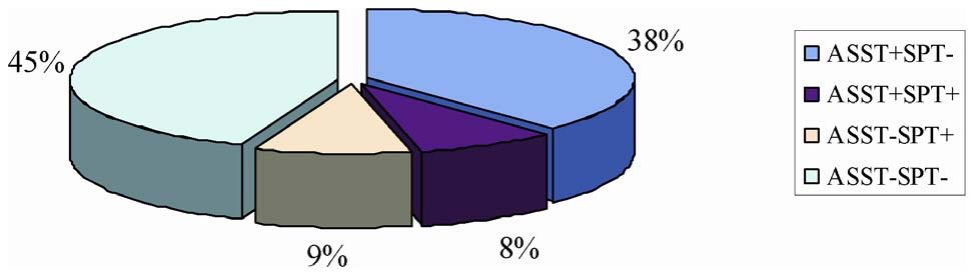
Distribution of subtype of chronic spontaneous urticaria based on the result of ASST and SPT in present study.

**Figure 3:**
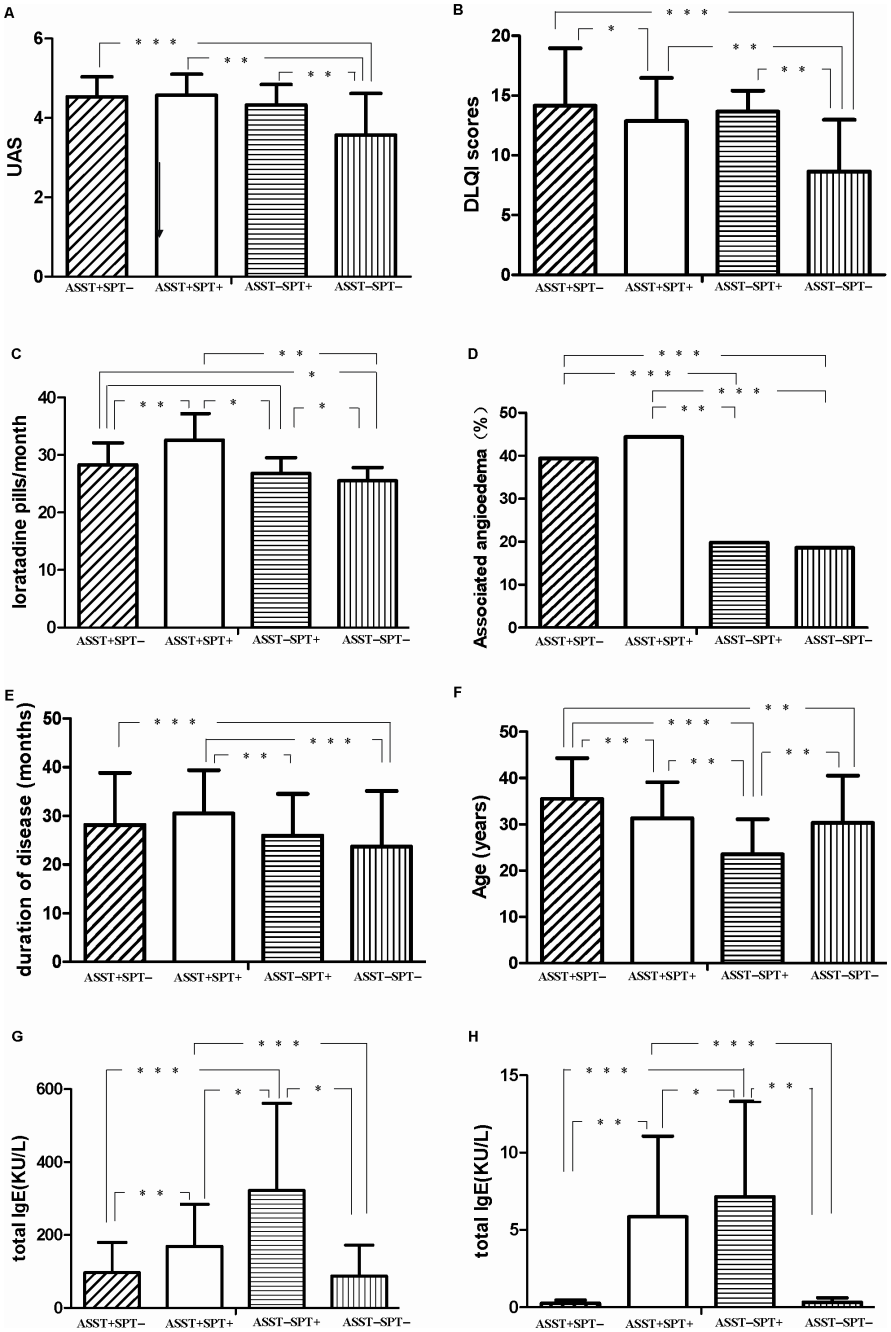
Comparison of UAS (A), DLQI (B), total required loratadine every month (C), percentages of associated angioedema (D), duration of disease (F), mean age (F), total IgE (G) and HDM-sIgE (H) among patients with ASST+SPT−, patients with ASST+SPT+, patients with ASST−SPT+ and patients with ASST−SPT−, respectively. *P<0.05. **P<0.01. ***P<0.0001.

To investigate whether different cutaneous reactions to autologous serum and/or HDM were associated with certain clinical features, patients were categorized into four groups based on the results of ASST and SPT to HDM, as shown in Fig. 2. The one-way analysis of variance (one-way ANOVA) and Tukey's post-hoc test were performed to detect statistical differences of clinical and laboratory parameters among subgroups and results of the test for interaction on the subgroup analyses were listed in Table 2. Results showed that patients that were positive for ASST and/or SPT had higher scores of disease activity and DLQI, compared with patients with negative ASST and SPT, as shown in Fig. 3A and Fig. 3B. These patients also had higher doses of total required antihistamine medication every month (Fig. 3C) and higher frequencies of angioedema (*P*<0.0001) (Fig. 3D). Durations of disease in patients with positive ASST and/or SPT were significantly longer than those in patients with negative ASST and SPT (Fig. 3E). The mean age of ASST(−)SPT(+) group was 23.5±7.6 years (ranged 3–53 years) and significantly smaller than that in the ASST(+)SPT(−) group (35.5±8.8 (ranged 15–62)) (Fig. 3F). Moreover, the serum levels of total IgE and HDM-specific IgE in subgroups with ASST(+)SPT(+) and ASST(−)SPT(+) are higher than those in other two subgroups (Fig. 3G and Fig. 3H).

**Table 2:** One-way analysis of variances of clinical and laboratory data among different subgroups based on the result of ASST and SPT.

Source of variation	R-squared	F	P value
Mean age	0.1289	42.34	<0.0001
duration of disease	0.04804	14.43	<0.0001
UAS	0.2561	98.47	<0.0001
DLQI	0.2705	106.0	<0.0001
loratadine pills per month	0.2883	115.9	<0.0001
T-IgE	0.2787	110.5	<0.0001
HDM-sIgE	0.4970	282.6	<0.0001

## Discussion

Positivity to autologous serum test is found in one-third CU cases, known as autoreactive chronic urticaria (ACU), in which IgG antibodies play an important part by interacting with the α chain of the high-affinity IgE receptor (FcεRIα) [6], [7], [8], [10]. Although the prevalence and significance of ASST in CU has been widely investigated, there is considerable variation in the positive rate of ASST in different studies [4], [7], [8], [9], [10], [11], [12]. The present study investigated the prevalence of HDM sensitization and the prevalence of ASST in patients with CSU. Our results showed 46.3% of patients had various degrees of positive ASST reaction, and 17.8% of patients showed skin prick test sensitivity to HDM, which are in line with earlier reports [13], [14], [15], [17], [18], [19].

As an inducing or deteriorating factor, aeroallergen, mainly due to house dust mites, may contribute to allergic diseases. However, the significance of mite sensitization in the pathogenesis of CSU is still in dispute [13], [14], [15], [17], [18], [19], [25]. Theoretically, it is possible that HDM induce urticarial reactions *via* entering the body through inhalation or through the epidermis [20], [26]. Previous studies, and our clinical practice, have revealed that patients with evaluated HDM-sIgE antibodies in serum typically accompany positive SPT against HDM. Additionally, correlation between skin test reactivity and *in vitro* mite-induced histamine release from leukocytes has been demonstrated [15], [20]. Furthermore, avoidance of mites can partly relieve symptoms in small subgroup of patients in clinical practices, and remission of symptoms of urticaria after HDM immunotherapy in patients with respiratory house dust mite allergy and chronic urticaria, has been observed [26].

In this study, 23 patients showed positive SPT with a higher reaction degree (3+) and evaluated sIgE against HDM. Their urticaria symptoms became worse when they remained in a room with a high density of HDM, and were markedly ameliorated in an HDM-free environment. Among these 23 patients, there were two patients that had a personal history of atopic diseases, and five cases had allergic rhinitis and/or asthma. Furthermore, in a previous open label trial, we demonstrated that specific immunotherapy with a standardized house dust mite vaccine significantly ameliorate symptoms and reduce the relapse rate in chronic spontaneous urticaria patients with positive IgE directed to HDM [21].

Besides the female predominance in ASST positive patients, which was in line with most previous studies [8], [9], [10], [11], we also found that the prevalence of HDM sensitivity in positive-ASST subgroups of patients was significantly lower than that in negative-ASST subgroups. Further, HDM sensitivity was more common in young patients aged 20 or less. Whether this could be due to different backgrounds of atopic and autoreactive diathesis among various patient groups needs further investigation.

Treatment of CSU is difficult because in most of cases, the underlying cause is unclear [1], [2]. The current clinical classification is based on disease duration and frequency, which does not provide much help towards elucidating a specific treatment [1], [2]. We proposed a new classification for CSU in this study on the basis of revised nomenclature for allergy [27], as presented in Figure 2. The first subtype (9%) is CSU patients with environmental allergen sensitivity, characterized with positive SPT directed to environmental allergens, ie. HDM in present study, but with negative ASST. Type I allergy is a rare cause of chronic spontaneous urticaria in patients who present with daily or almost daily symptoms, but must be considered in chronic spontaneous urticaria patients with intermittent symptoms [1], [14], [15], [17], [18], [19], [20], [21]. In our study, most patients with positive SPT but negative ASST presented intermittent urticarial symtoms and higher titre of HDM-specific IgE than other groups [21]. However, whether related allergen is likely to be one of the major causes of this subtype of CSU need further investigation [20], [21], [28]. The second subtype (38%) is chronic autoreactive urticaria, in which patients present with negative SPT but positive ASST. In this subtype of CSU, the activation of mast cells and basophiles may primarily caused by autoantibodies against the high-affinity receptor of IgE, IgE itself, or other histamine release factors (HRFs) from serum [7], [8], [9], [10], [28], [29], [30]. The third subtype (8%) involves both autoreactivity and allergen sensitivity, which presented with positive SPT to environmental allergen and positive ASST. Higher disease activity scores can be observed in this subtype, thus, high doses of anti-histamines treatment are required. The fourth subtype (45%) of CSU is presented with negative ASST and negative SPT. This subtype could be considered as the true idiopathic urticaria at present [4].

Present study showed that there were significant differences in mean age, UAS,DLQI, duration of diseases, frequency of associated angioedema, total required loratadine every month, and the serum levels of total & specific IgE, among above four subtypes, which might be helpful for subtype-specific therapeutic schedule. Based on this proposed classification for CSU, we have evaluated the clinical outcomes of specific immunotherapy (SIT) with standardized HDM vaccine on CSU patients with positive SPT to HDM and evaluated specific IgE to HDM, but negative ASST, for at least one year. Results showed SIT could not only reduced the total required anti-histamines, but also decreased the serum level of specific IgE after 6 months of therapy [21]. Recent studies have shown patients with positive ASST exhibited improvement of urticarial symptoms and quality of life, and reduced requirement for antihistaminic medication after injections of autologus whole blood [31], [32], [33], also in our recent similar study [34]. These studies suggest that subtype-specific treatment may achieve better clinical outcomes.

In conclusion, this study provided a profile of ASST and HDM sensitization in Chinese patients with CSU. Patients with different ASST and SPT phenotypes showed different disease activities and potentially require different management.

## References

[1] Zuberbier T, Bindslev-Jensen C, Canonica W, Grattan CE, Greaves MW, et al (2006) EAACI/GA2LEN/EDF.EAACI/GA2LEN/EDF guideline: definition, classification and diagnosis of urticaria. Allergy 61: 316–320. 16436140 10.1111/j.1398-9995.2005.00964.x

[2] Maurer M, Weller K, Bindslev-Jensen C, Giménez-Arnau A, Bousquet PJ, et al (2011) Unmet clinical needs in chronic spontaneous urticaria. A GA^2^LEN task force report.Allergy 66: 317–330. 10.1111/j.1398-9995.2010.02496.x 21083565

[3] Najib U, Sheikh J (2009) The spectrum of chronic urticaria. Allergy Asthma Proc 30: 1–10. 19331714 10.2500/aap.2009.30.3191

[4] Kaplan AP, Greaves M (2009) Pathogenesis of chronic urticaria. Clin Exp Allergy 39: 777–787. 19400905 10.1111/j.1365-2222.2009.03256.x

[5] Wedi B (2008) Urticaria. J Dtsch Dermatol Ges 6: 306–317. 18377563 10.1111/j.1610-0387.2008.06661.x

[6] Młynek A, Maurer M, Zalewska A (2008) Update on chronic urticaria: focusing on mechanisms. Curr Opin Allergy Clin Immunol 8: 433–437. 18769197 10.1097/ACI.0b013e32830f9119

[7] Boguniewicz M (2008) The autoimmune nature of chronic urticaria. Allergy Asthma Proc 29: 433–438. 18926050 10.2500/aap.2008.29.3148

[8] Sabroe RA, Grattan CE, Francis DM, Barr RM, Kobza Black A, et al (1999) The autologous serum shin test for autoantibodies in chronic idiopathic urticaria. Br J Dermatol 140: 446–452. 10233264 10.1046/j.1365-2133.1999.02707.x

[9] Konstantinou GN, Asero R, Maurer M, Sabroe RA, Schmid-Grendelmeier P, et al (2009) EAACI/GA^2^LEN task force consensus report: the autologous serum skin test in urticaria. Allergy 64: 1256–1268. 19650847 10.1111/j.1398-9995.2009.02132.x

[10] Sheikh J (2005) Autoantibodies to the high-affinity IgE receptor in chronic urticaria: how important are they? Curr Opin Allergy Clin Immunol 5: 403–437. 16131914 10.1097/01.all.0000182540.45348.bc

[11] Buss YA, Garrelfs UC, Sticherling M (2007) Chronic urticaria: which clinical parameters are pathogenetically relevant? A retrospective investigation of 339 patients. J Dtsch Dermatol Ges 5: 22–29. 17229201 10.1111/j.1610-0387.2007.06194.x

[12] Yıldız H, Karabudak O, Doğan B, Harmanyeri Y (2011) Evaluation of autologous plasma skin test in patients with chronic idiopathic urticaria. Br J Dermatol 165: 1205–1209. 21910697 10.1111/j.1365-2133.2011.10582.x

[13] Ronchetti R, Haluszka J, Martella S, Falasca C, Guglielmi F, et al (2003) Skin reactivity to histamine and to allergens in unselected 9-year-old children living in Poland and Italy. Pediatr Allergy Immunol 14: 201–206. 12787299 10.1034/j.1399-3038.2003.00027.x

[14] Caliskaner Z, Ozturk S, Turan M, Karaayvaz M (2004) Skin test positivity to aeroallergens in the patients with chronic urticaria without allergic respiratory disease. J Investig Allergol Clin Immunol 14: 50–54. 15160442

[15] Kulthanan K, Jiamton S, Rutnin NO, Insawang M, Pinkaew S (2008) Prevalence and relevance of the positivity of skin prick testing in patients with chronic urticaria. J Dermatol 35: 330–335. 18578709 10.1111/j.1346-8138.2008.00477.x

[16] Daschner A, Rodero M, De Frutos C, Valls A, Cuéllar C (2010) Chronic urticaria is associated with a differential helminth-arthropod-related atopy phenotype. J Dermatol 37: 780–785. 20883361 10.1111/j.1346-8138.2010.00869.x

[17] Caliskaner Z, Ozturk S, Turan M, Karaayvaz M (2004) Skin test positivity to aeroallergens in the patients with chronic urticaria without allergic respiratory disease. J Investig Allergol Clin Immunol 14: 50–54. 15160442

[18] Mahesh PA, Kushalappa PA, Holla AD, Vedanthan PK (2005) House dust mite sensitivity is a factor in chronic urticaria. Indian J Dermatol Venereol Leprol 71: 99–101. 16394382 10.4103/0378-6323.13993

[19] Kulthanan K, Wachirakaphan C (2008) Prevalence and clinical characteristics of chronic urticaria and positive skin prick testing to mites. Acta Derm Venereol 88: 584–588. 19002343 10.2340/00015555-0546

[20] Numata T, Yamamoto S, Yamura T (1980) The role of mite, house dust and candida allergens in chronic urticaria. J Dermatol 7: 197–202. 6997350 10.1111/j.1346-8138.1980.tb03532.x

[21] Song ZQ, Bao CN, Zhai ZF, Zhong H, Zhong BY, et al (2008) Effect of specific immunotherapy in chronic urticaria patients with IgE antibody against house-dust mite. J Clin Dermatol 37: 809–811.

[22] Zhou X, Huang X, Feng L, Song Z, Zhong H (2011) Comparison among various positive controls in autologous serum skin test and its clinical significances. J Clin Dermatol 40: 224–225.

[23] Jariwala SP, Moday H, de Asis ML, Fodeman J, Hudes G, et al (2009) The Urticaria Severity Score: a sensitive questionnaire/index for monitoring response to therapy in patients with chronic urticaria. Ann Allergy Asthma Immuno 102: 475–482. 10.1016/S1081-1206(10)60120-2 19558005

[24] Lennox RD, Leahy MJ (2004) Validation of the Dermatology Life Quality Index as an outcome measure for urticaria-related quality of life. Ann Allergy Asthma Immunol 93: 142–146. 15328673 10.1016/S1081-1206(10)61466-4

[25] Augey F, Gunera-Saad N, Bensaid B, Nosbaum A, Berard F, et al (2011) Chronic spontaneous urticaria is not an allergic disease. Eur J Dermatol 21: 349–353. 21616748 10.1684/ejd.2011.1285

[26] Kasperska-Zajac A, Brzoza Z (2009) Remission of chronic urticaria in the course of house dust mite immunotherapy–mere coincidence or something more to it? Vaccine 27: 7240–7241. 19737528 10.1016/j.vaccine.2009.08.076

[27] Johansson SG, Hourihane JO, Bousquet J (2001) A revised nomenclature for allergy. An EAACI position statement from the EAACI nomenclature task force. Allergy 56: 813–824. 11551246 10.1034/j.1398-9995.2001.t01-1-00001.x

[28] Irinyi B, Széles G, Gyimesi E, Tumpek J, Herédi E, et al (2007) Clinical and laboratory examinations in the subgroups of chronic urticaria. Int Arch Allergy Immunol 144: 217–225. 17579280 10.1159/000103995

[29] Sabroe RA, Seed PT, Francis DM, Barr RM, Black AK, et al (1999) Chronic idiopathic urticaria: comparison of the clinical features of patients with and without anti-FcepsilonRI or anti-IgE autoantibodies. J Am Acad Dermatol 40: 443–450. 10071316 10.1016/s0190-9622(99)70495-0

[30] Toubi E, Kessel A, Avshovich N, Bamberger E, Sabo E, et al (2004) Clinical and laboratory parameters in predicting chronic urticaria duration: a prospective study of 139 patients. Allergy 59: 869–873. 15230821 10.1111/j.1398-9995.2004.00473.x

[31] Staubach P, Onnen K, Vonend A, Metz M, Siebenhaar F, et al (2006) Autologous whole blood injections to patients with chronic urticaria and a positive autologous serum skin test: a placebo-controlled trial. Dermatology 212: 150–159. 16484822 10.1159/000090656

[32] Bajaj AK, Saraswat A, Upadhyay A, Damisetty R, Dhar S (2008) Autologous serum therapy in chronic urticaria: Old wine in a new bottle. Indian J Dermatol Venereol Lepro 74: 109–113. 10.4103/0378-6323.39691 18388366

[33] Kocatürk E, Aktaş S, Türkoğlu Z, Kavala M, Zindanci I, et al (2011) Autologous whole blood and autologous serum injections are equally effective as placebo injections in reducing disease activity in patients with chronic spontaneous urticaria: a placebo controlled, randomized, single-blind study. J Dermatolog Treat 31: 1–7. 10.3109/09546634.2011.593485 21801101

[34] Chen S, Zhai Z, Song Z, Feng L, Huang X, et al (2012) Effects of autologus whole blood injections to patients with chronic spontaneous urticaria with positive autologous serum skin test. Chin J Dermatol 45: 12–15.

